# Enzymatic Hydrolytic Resolution of Racemic Ibuprofen Ethyl Ester Using an Ionic Liquid as Cosolvent

**DOI:** 10.3390/molecules21070905

**Published:** 2016-07-13

**Authors:** Tao Wei, Kunpeng Yang, Bing Bai, Jie Zang, Xuan Yu, Duobin Mao

**Affiliations:** 1 School of Food and Biological Engineering, Zhengzhou University of Light Industry, 5 Dongfeng Rd., Zhengzhou 450002, China; weit8008@zzuli.edu.cn (T.W.); 15238358950@163.com (K.Y.); baibing82@163.com (B.B.); pds0188@126.com (J.Z.); 15038262577@126.com (X.Y.); 2 Collaborative Innovation Center of Food Production and Safety, 136 Science Avenue, Zhengzhou 450002, China

**Keywords:** cosolvent, enzymatic, ionic liquid, racemic ibuprofen, (*S*)-ibuprofen

## Abstract

The aim of this study was to develop an ionic liquid (IL) system for the enzymatic resolution of racemic ibuprofen ethyl ester to produce (*S*)-ibuprofen. Nineteen ILs were selected for use in buffer systems to investigate the effects of ILs as cosolvents for the production of (*S*)-ibuprofen using thermostable esterase (EST10) from *Thermotoga maritima*. Analysis of the catalytic efficiency and conformation of EST10 showed that [OmPy][BF_4_] was the best medium for the EST10-catalyzed production of (*S*)-ibuprofen. The maximum degree of conversion degree (47.4%), enantiomeric excess of (*S*)-ibuprofen (96.6%) and enantiomeric ratio of EST10 (177.0) were achieved with an EST10 concentration of 15 mg/mL, racemic ibuprofen ethyl ester concentration of 150 mM, at 75 °C , with a reaction time of 10 h. The reaction time needed to achieve the highest yield of (*S*)-ibuprofen was decreased from 24 h to 10 h. These results are relevant to the proposed application of ILs as solvents for the EST10-catalyzed production of (*S*)-ibuprofen.

## 1. Introduction

Ibuprofen (2-(4-isobutylphenyl)propionic acid) is an important non-steroidal anti-inflammatory drug that is widely used for the treatment of headaches, rheumatoid arthritis, and muscular strains [1,2,3]. Commercially available ibuprofen is usually a racemic mixture, as the racemate is easily obtained through chemical synthesis. However, (*S*)-ibuprofen is about 160 times more active than (*R*)-ibuprofen. Moreover, the presence of (*R*)-ibuprofen can cause serious side effects, such as gastrointestinal tract irritation, and disruption of normal lipid metabolism and membrane function [4,5]. Therefore, much effort has been therefore devoted to the production of pure (*S*)-ibuprofen. Among the approaches for obtaining a single enantiomer of ibuprofen, hydrolase-catalyzed enzymatic kinetic resolution has attracted considerable interest because of its enantioselectivity, regioselectivity, and substrate specificity under mild conditions [6,7].

Ionic liquids (ILs) are salts made up of ions that remain liquid at room temperature or temperatures below 100 °C. ILs have unique properties that make them useful as green solvents for many biocatalytic reactions [8,9]. They are non-volatile, non-flammable, and have excellent chemical and thermal stability, which can provide enzymes with excellent levels of activity, stability, and stereoselectivity [10,11]. For enzyme catalysis, the activity and stability of enzymes are closely related to the physicalchemical properties of ILs (e.g., polarity, viscosity, hydrogen bond basicity, hydrophobicity and anion concentration) [12]. The use of enzymes, especially lipases, in ionic liquids, has demonstrated many advantages, such as high conversion rates, good enantioselectivity, better recoverability and recyclability. For example, the stability of lipases such as CaLB [13,14,15,16] and *Pseudomonas cepacia* lipase (PcL) [15,17] are catalytically active in imidazolium and pyridinium ILs containing BF_4_^−^, PF_6_^−^ and Tf_2_N^−^ [14]. [OmPy][BF_4_] provide high enantioselectivity to hesperidinase when used as co-solvent in the enzymatic conversion of rutin to isoquercitrin. The reaction time decreased 0.33-fold, and the rutin conversion and isoquercitrin yield increased 1.67-fold and 2.33-fold, respectively, in [OmPy][BF_4_]_-_containing buffer systems. Besides acting as a reaction media, ILs can also be used to pre-treat enzyme. There are many literature data on enhanced the activity and stability of enzyme by coating it with ILs which had been used for transesterification, esterification and hydrolysis reaction with higher enantioselecticity and yields [12,18].

**Scheme 1:**
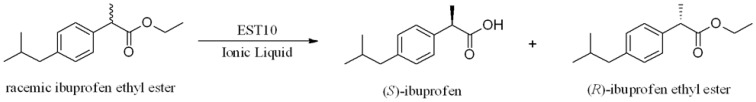
EST10-catalyzed enantioselective hydrolysis of racemic eaters.

Several thermophilic esterases have been reported to have the ability to resolve mixtures of chiral esters. So far only one thermophilic esterase APE147 from archaeon *Aeropyrum pernix* K1 with a strict stereoselectivity toward (*S*)-ibuprofen was identified and characterized. The enantioselectivity of EST10 was higher than that of APE147, which showed *ee*_p_ of 99% and *E* value of 38.1 at 57% conversion in 96 h [19]. In an earlier study, the thermostable esterase (EST10) from *Thermotoga maritima* was identified and characterized [20], and EST10 showed high thermostability and gave a high hydrolytic resolution of racemic ketoprofen ethyl ester compared with other esterases. However, EST10 exhibited low enantioselectivity toward (*R*,*S*)-racemic ibuprofen ethyl ester (enantioselectivity, *E*-value < 10) (Scheme 1) in both aqueous and organic solvents [20]. The objective of this study was to develop an IL system for high-yield enzymatic production (EST-catalyzed) of (*S*)-ibuprofen racemic ibuprofen ethyl ester. The effects of different ILs and reaction parameters on the enantioselectivity toward racemic ibuprofen ethyl ester were also investigated.

## 2. Results and Discussion

### 2.1. Effects of ILs

The effects of 19 different ILs were evaluated for the production of (*S*)-ibuprofen from the EST-catalyzed hydrolysis of racemic ibuprofen ethyl ester. Table 1 shows that the structure of the IL had a large impact on the degree of conversion (*X* = 2.3%–46.5%), enantiomeric excess of the product (*ee*_p_ = 1.4%–96.2%), and enantiomeric ratio (*E* = 1.0–134.7) of EST10. The catalytic efficiencies of the different anions were in the following order: [BF_4_]^−^ ˃ [Tf_2_N]^−^ ˃ [PF_6_]^−^ ˃ [NO_3_]^−^ ˃ [CH_3_COO]^−^ ˃ [TfO]^−^ ˃ [CH_3_SO_3_]^−^ ˃ [Cl]^−^. The highest enantioselectivity for the hydrolysis of ibuprofen ethyl ester was achieved using [OmPy][BF_4_] in the buffer system, giving *X* 46.5%, *ee*_p_ of 96.2%, and *E* of 134.7. The (*S*)-ibuprofen yields for ILs containing [BF_4_]^−^, [Tf_2_N]^−^, and [PF_6_]^−^ anions were higher than those observed for the ILs containing [NO_3_]^−^, [CH_3_COO]^−^, [TfO]^−^, [Cl]^−^, and [CH_3_SO_3_]^−^ anions. ILs containing structurally different anions could potentially interact with protein molecule in different ways, including hydrogen bonding, van der Waals, ionic and dipolar interactions. The hydrogen bond basicity and nucleophilicity of anions have been found to affect the enzyme activity and stability in IL-based solvent media [12,21]. Anions having lower hydrogen bond basicity and nucleophilicity are found to be enzyme-compatible, because low hydrogen bond basicity and nucleophilicity minimize their interference with the internal hydrogen bonds and interaction with the positively charged sites in the enzyme and hence reduce the tendency to change the conformation of the enzyme’s structure [12,21,22,23]. This difference in (*S*)-ibuprofen yields could be attributed to the fact that ILs containing [NO_3_]^−^, [CH_3_COO]^−^, [TfO]^−^, [Cl]^−^, and [CH_3_SO_3_]^−^ anions have higher hydrogen bond basicity and nucleophilicity than those containing [BF_4_]^−^, [Tf_2_N]^−^, and [PF_6_]^−^ anions. This difference in hydrogen bond basicity and nucleophilicity could result in a change in the conformation of the enzyme, which could lead to a loss of activity [24,25]. Therefore, the EST-catalyzed synthesis of (*S*)-ibuprofen would occur much more effectively in enzymatic media containing ILs with weakly coordinating anions such as [BF_4_]^−^, [Tf_2_N]^−^, and [PF_6_]^−^. This conclusion is in agreement with the results of several previous studies using ILs in the reaction media [26,27]. A comparison of the enantioselectivity achieved with ILs containing the same anions (i.e., [BF_4_]^−^ and [PF_6_]^−^ and different cations (i.e., [Emim]^+^, [Bmim]^+^, [Hmim]^+^, [Omim]^+^ and [Dmim]^+^) revealed that the (*S*)-ibuprofen yield increased as the length of the alkyl chain attached to the imidazolium ring of the IL increased (Table 1). This increase in yield with increasing chain length could be attributed to the cations becoming increasingly hydrophobic with increasing chain length, which would lead to a reduction in the water-stripping capacity of the ILs and the occurrence of unwanted protein–ion interactions [28]. Our results show that [OmPy][BF_4_] is the most suitable of all of the ILs tested in the current study as a reaction medium for the EST-catalyzed production of (*S*)-ibuprofen.

### 2.2. Conformational Studies of EST

Conformational changes in the EST enzyme could cause the enantioselectivity differences observed with the different ILs. Circular dichroism (CD) has been widely used to gather information about the structural features of proteins. CD spectroscopy was conducted to determine the effects of the different ILs on the secondary structure of the EST enzyme. After treatment with selected ILs containing different anions (e.g., [PF_6_]^−^ and [BF_4_]^−^) and cations (e.g., [Emim]^+^, [Bmim]^+^, [Hmim]^+^, [Omim]^+^, and [Dmim]^+^), there were large changes in the secondary structural elements of the EST enzyme, including a decrease in the α-helical content and an increase in the β-sheet content (Table 2). This result indicated that the observed decrease in the α-helical content of the enzyme would most likely have an impact on the active site of EST, which would lead to an increase in its enantioselectivity. Similar results have been reported for lipases from *Candida rugosa*, *Burkholderia cepacia*, *Chromobacterium viscosum*, and *Thermomyces lanuginose* in non-aqueous media, and these confirm that higher catalytic activity can be related to a decrease in the α-helical content of the enzyme [27,29,30]. Further studies are required to develop a deeper understanding of the relationship between the enhanced enantioselectivity of EST and changes to its structure.

### 2.3. Effects of IL Concentration

**Figure 1:**
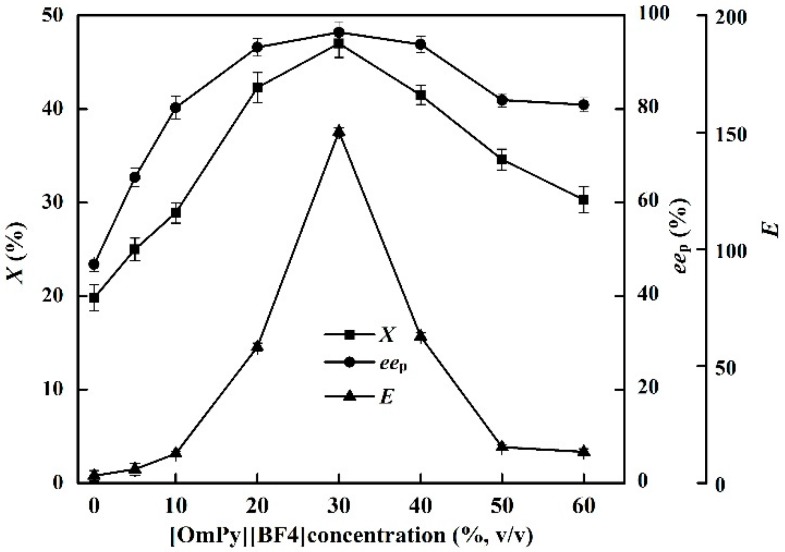
Effect of IL concentrations on the conversion degree, enantiomeric excess of product and enantioselectivity of EST10 using [OmPy][BF_4_] as a co-solvent in the presence of enzyme EST10 (15 mg/mL) at 75 °C for 10 h. The concentration of racemic ibuprofen ethyl ester was 2.5 mg/mL. The values are means of three independent experiments.

The effects of [OmPy][BF_4_] concentration on the *X*, *ee*_p_ and *E* of the enzyme in the EST-catalyzed production of (*S*)-ibuprofen were investigated (Figure 1). The IL concentration in the medium (0%–60%) greatly affected the catalytic efficiency of EST10. The highest *X* (47.0%) and *ee*_p_ (96.4%) were achieved at an IL concentration of 30% ([OmPy][BF_4_]/buffer, *v*/*v*). The *X* and *ee*_p_ decreased sharply as the IL concentration was increased from 30% to 60%. The highest *E* of EST10 (150.3) was also achieved in the [OmPy][BF_4_]–buffer (30:70, *v*/*v*) system. A higher concentration of IL not only increases the ionic strength in the enzymatic reaction medium, which might inactivate the enzyme, but also increases the viscosity of the reaction mixture, which limits diffusion of reaction substrates to the enzyme active site [31].

### 2.4. Effects of Time

**Figure 2:**
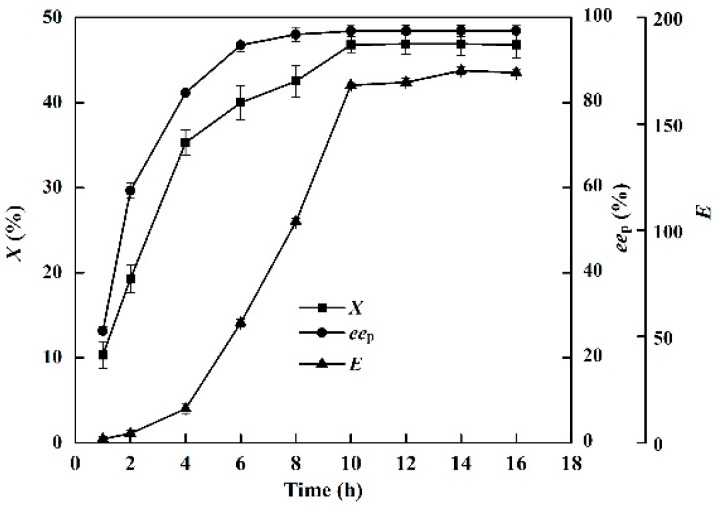
Effect of reaction time (from 0 to 16 h) on the conversion degree, enantiomeric excess of product and enantioselectivity of EST10 using [OmPy][BF_4_] as a co-solvent in the presence of enzyme EST10 (15 mg/mL) at 75 °C. The concentration of racemic ibuprofen ethyl ester was 2.5 mg/mL. The values are means of three independent experiments.

The effects of reaction time (0–16 h) on the conversion degree, enantiomeric excess of product and enantioselectivity of the enzyme in the EST-catalyzed production of (*S*)-ibuprofen are shown in Figure 2. After an enzymatic reaction time of 10 h, no significant improvement in the production of (*S*)-ibuprofen was observed. The *X*, *ee*_p_ and *E* of EST10 were 46.8%, 96.8%, and 168.2, respectively. The reaction time of industrial procedure of (*S*)-ibuprofen (˂10 h), such as enantioselective crystallization and chemical asymmetric synthesis, is shorter than that of the enzymatic resolution. However, almost all industrial methods involves a complicated synthesis that requires harsh separation techniques [32,33]. Enzymatic hydrolysis of racemic ibuprofen ethyl ester usually requires a long reaction time (over 24 h) to achieve the maximum yield in both aqueous and organic solvents [31,34,35]. However, the maximum conversion degree, enantiomeric excess of product and enantioselectivity of enzyme were achieved after only 10 h in the [OmPy][BF_4_]–buffer (30:70, *v*/*v*) system.

### 2.5. Effects of Temperature

**Figure 3:**
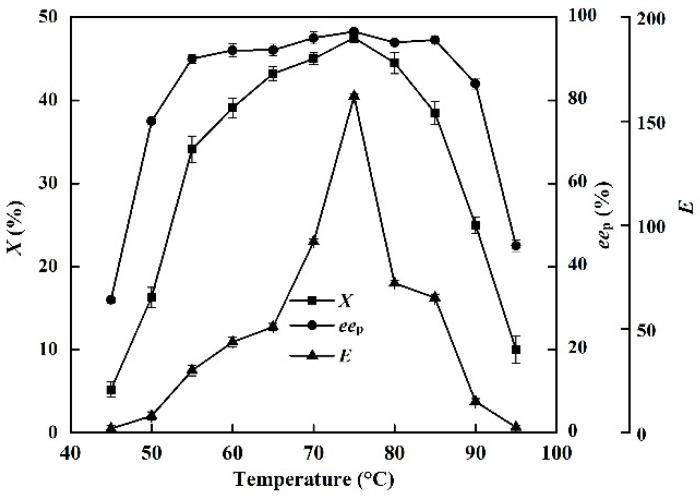
Effect of reaction temperature (from 45 °C to 95 °C) on the conversion degree, enantiomeric excess of product and enantioselectivity of EST10 using [OmPy][BF_4_] as a co-solvent in the presence of enzyme EST10 (15 mg/mL). The concentration of racemic ibuprofen ethyl ester was 2.5 mg/mL. The values are means of three independent experiments.

The effects of temperature (45–95 °C) on *X*, *ee*_p_, and *E* of the enzyme in the EST-catalyzed production of (*S*)-ibuprofen were investigated (Figure 3). 

When the reaction was conducted in the [OmPy][BF_4_]–buffer (30:70, *v*/*v*) system, the *X* and *ee*_p_ increased sharply when the temperature was increased from 45 to 75°C. The optimum reaction temperature was 75 °C (Figure 3). When the reaction was conducted in the [OmPy][BF_4_]–buffer (30:70, *v*/*v*) system, the conversion degree and enantiomeric excess of product increased sharply with increasing temperature from 45 to 75 °C, with the optimum reaction temperature being 75 °C (Figure 3). This is slightly higher than that used in solvent-free systems (70 °C) [20].

### 2.6. Effects of pH

**Figure 4:**
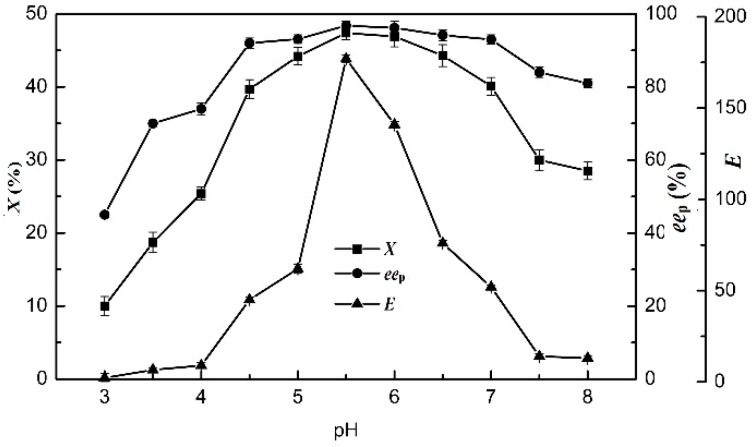
Effect of pH (from 3 to 8) on the degree of conversion, enantiomeric excess of product and enantioselectivity of EST10 using [OmPy][BF_4_] as a co-solvent in the presence of enzyme EST10 (15 mg/mL) at 75 °C. The concentration of racemic ibuprofen ethyl ester was 2.5 mg/mL. The values are means of three independent experiments.

The effect of pH on the enzymatic hydrolysis of racemic ibuprofen ethyl ester was examined at 75 °C from pH 3.0 to 8.0 (Figure 4). When the pH value was adjusted to 5.5 in the [OmPy][BF_4_]–buffer (30:70, *v*/*v*) system, the enzymatic hydrolysis of racemic ibuprofen ethyl ester reached the highest yield of (*S*)-ibuprofen. The results of *X*, *ee*_p_ and *E* were 47.4%, 96.8%, and 177, respectively. The optimal pH value of the buffer solution in the 30% (*v*/*v*) [OmPy][BF_4_]–containing system was 5.5. These results demonstrated that the IL co-solvents did not change the optimal pH values of EST, which was similar to that of EST in an aqueous buffer medium (5.5).

### 2.7. Effects of Substrate Concentration

**Figure 5:**
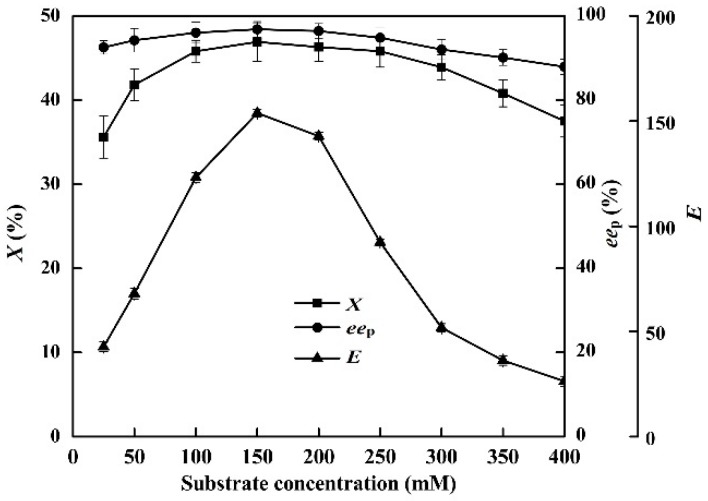
Effect of ibuprofen ethyl ester concentration on the conversion degree, enantiomeric excess of product and enantioselectivity of EST10 using [OmPy][BF_4_] as a co-solvent in the presence of enzyme EST10 (15 mg/mL) at 75 °C for 10 h. The values are means of three independent experiments.

The effects of substrate concentration (25–400 mM) on the enzymatic hydrolysis of racemic ibuprofen ethyl ester were investigated (Figure 5).

In the [OmPy][BF_4_]–buffer (30:70, *v*/*v*) system, the highest enantioselectivity for the hydrolysis of ibuprofen ethyl ester (X = 46.9%, *ee*_p_ = 96.8%, and *E* = 153.8) was achieved with 150 mM of racemic ibuprofen ethyl ester. Any further increase or decrease in the substrate concentration resulted in a decrease in the (*S*)-ibuprofen yield. This indicates that increasing the substrate concentration (from 25 to 150 mM) facilitates contact between the enzyme and the substrate in the [OmPy][BF_4_]–buffer (30:70, *v*/*v*) system.

### 2.8. Effects of Enzyme Concentration

**Figure 6:**
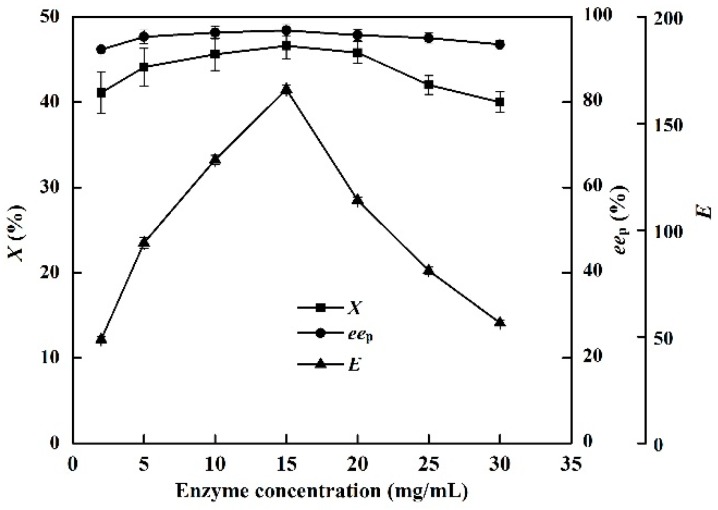
Effect of enzyme concentration on the conversion degree, enantiomeric excess of product and enantioselectivity of EST10 using [OmPy][BF_4_] as a co-solvent at 75 °C for 10 h. The concentration of racemic ibuprofen ethyl ester was 2.5 mg/mL. The values are means of three independent experiments.

The effects of enzyme concentration (0.2–5 U/mL) on *X*, *ee*_p_, and *E* in the EST-catalyzed production of (*S*)-ibuprofen were investigated. The *c* and *ee*_p_ increased considerably with increasing enzyme concentration (from 0.2 to 3.0 U/mL, Figure 6). The highest *X* (46.8%) and *ee*_p_ of (*S*)-ibuprofen (96.9%) were obtained at an enzyme concentration of 3 U/mL. However, the presence of excess catalyst (>3 U/mL) could result in enzyme agglomeration and diffusion problems, which could decrease the reaction efficiency [36]. An EST loading of 3 U/mL provided good catalytic efficiency in the [OmPy][BF_4_]–buffer (30:70, *v*/*v*) system.

## 3. Experimental Section

### 3.1. Chemicals

Nineteen ILs, including [Bmim][CH_3_SO_3_], [Omim][CH_3_SO_3_], [Bmim][Cl], [Emim][Cl], [Emim][Cl], [Emim][TfO], [Bmim][TfO], [Bmim][BF_4_], [Emim][BF_4_], [Omim][BF_4_], [OmPy][BF_4_], [Bmim][PF_6_], [Emim][PF_6_], [Hmim][PF_6_], [Omim][PF_6_], [Dmim][PF_6_], [Bmim][Tf_2_N], [Bmim][CH_3_COO], and [Bmim][NO_3_], with purities ≥ 99.9%, were purchased from Shanghai Cheng Jie Chemical Co. Ltd (Shanghai, China). (*R*,*S*)-Ibuprofen ethyl ester, (*S*)-ibuprofen and (*R*)-ibuprofen were purchased from J & K Scientific Ltd. (Beijing, China). All other chemicals were of the highest grade available and obtained from Sangon (Shanghai, China). The EST10 enzyme was purified according to a previously reported procedure [20].

### 3.2. Enzyme Assay

The EST10 enzyme was used with 19 different ILs containing six different cations and eight different anions to investigate their effects on the enantioselectivity and enzymatic production of (*S*)-ibuprofen. The standard reaction was carried out in a 10-mL round-bottom flask containing 0.3 mL of IL, 10 mg of the EST10 enzyme, 25 mg of racemic ibuprofen ethyl ester, and 0.7 mL of 50 mM sodium acetate buffer (pH 5.5). The resulting mixture was shaken (180 rpm) at 65 °C for 10 h. These conditions were used for all experiments, except when otherwise stated. The effects of IL concentration, reaction time, reaction temperature, and EST concentration on the production of (*S*)-ibuprofen were investigated.

### 3.3. HPLC Analysis

Reactants and products were analyzed by HPLC using a chiracel QJ-Hcolumn (25 cm × 4.6 cm, Daicel Chemical Industries Ltd., Tokyo, Japan). The samples were eluted with *n*-hexane: 2-propanol:acetic acid (90:10:0.5, *v*/*v*/*v*) at a flow rate of 1.0 mL/min and detected at 254 nm. Racemic ibuprofen ethyl ester, (*R*)-ibuprofen, and (*S*)-ibuprofen were detected at retention times of 4.9, 13.0, and 18.6 min, respectively. The *E* was calculated from the *ee*_p_ and *X* according to the equations described by Chen et al. [37]:

(1) $$X\;(\%)=\frac{\left[(S)\text{-ibuprofen}]+[(R)\text{-ibuprofen}\right]}{\text{initial ibuprofen ethyl ester}}\times 100\%$$

(2) $${ee}_{p}\;(\%)=\frac{\left[(S)\text{-ibuprofen}]-[(R)\text{-ibuprofen}\right]}{\left[(S)\text{-ibuprofen}]+[(R)\text{-ibuprofen}\right]}\times 100\%$$

(3) $$E=\frac{\ln[1-X(1+{ee}_{p})]}{\ln[1-X(1-{ee}_{p})]}$$

### 3.4. CD Spectroscopy

CD spectra (190–250 nm) of the EST enzyme were recorded on a Chirascan spectropolarimeter (Applied Photophysics, Leatherhead, UK) at 25 °C using a 0.1-cm quartz cuvette. Measurements were conducted using 0.2 mg/mL solutions with or without an IL (0.1 M). The results of six spectra were average for each sample. The scan speed, response time, and bandwidth were 200 nm·min^−^^1^, 0.5 s, and 1 nm, respectively. The sample spectra were obtained by subtracting the spectrum of the appropriate blank media without EST from the experimental spectra. The secondary structures of EST determined from the CD spectra were analyzed using the K2D2 web server provided by EMBL (Heidelberg, Germany).

## 4. Conclusions

In this study, we investigated the enzymatic synthesis of (*S*)-ibuprofen in EST-catalyzed reaction systems, using 19 different ILs as cosolvents. A [OmPy][BF_4_]-buffer (30:70, *v*/*v*) system was used as the reaction medium for the enzymatic production of (*S*)-ibuprofen by the EST-catalyzed resolution of racemic ibuprofen ethyl ester. A high *X* (47.4%), *ee*_p_ of (*S*)-ibuprofen (96.6%), and *E* of EST10 (177.0) were achieved under the following optimum conditions: EST10 concentration 15 mg/mL, racemic ibuprofen ethyl ester concentration 150 mM, temperature 75 °C, with a reaction time of 10 h. The reaction time needed to achieve the highest yield of (*S*)-ibuprofen was decreased from 24 h to 10 h. These results show that [OmPy][BF_4_] is a promising cosolvent for the enzymatic production of (*S*)-ibuprofen via biotransformation.

## Figures and Tables

**Table 1:** Effect of ionic liquid on the enantioselective hydrolysis of (*R*,*S*)-racemic ibuprofen ethyl ester by EST10.

Ionic Liquids	X (%)	*ee*_p_ (%)	*E*
Control	19.5	45.2	2.95
[Emim][PF_6_]	25.3	75.4	9.1
[Bmim][PF_6_]	39.2	85.6	22.4
[Hmim][PF_6_]	42.1	91.2	43.3
[Omim][PF_6_]	43.2	93.5	63.5
[Dmim][PF_6_]	44.2	94.8	84.9
[Emim][BF_4_]	34.5	76.9	11.3
[Bmim][BF_4_]	40.6	87.6	27.8
[Omim][BF_4_]	44.9	92.3	56.9
[OmPy][BF_4_]	46.5	96.2	134.7
[Bmim][Tf_2_N]	44.6	95.2	94.5
[Emim][TfO]	10.2	35.0	2.2
[Bmim][TfO]	8.3	19.5	1.5
[Bmim][CH_3_SO]	4.3	1.4	1.0
[Omim][CH_3_SO]	5.8	4.5	1.1
[Bmim][Cl]	6.8	9.5	1.2
[Emim][Cl]	2.3	0.6	1.0
[Omim][Cl]	3.5	3.8	1.1
[Bmim][NO_3_]	34.1	82.2	15.5
[Bmim][CH_3_COO]	32.1	66.9	6.8

Values given are the average of three replicates.

**Table 2:** The secondary structure elements of EST in ILs by CD spectroscopy.

Ionic Liquids	α-Helix (%)	β-Sheet (%)	β-Turn (%)	Random Coil (%)
Control	21.5 ± 0.3	26.5 ± 0.5	35.2 ± 0.2	16.8 ± 0.2
[Emim][PF_6_]	21.3 ± 0.2	26.0 ± 0.4	36.3 ± 0.2	16.4 ± 0.1
[Bmim][PF_6_]	20.5 ± 0.2	27.8 ± 0.3	34.7 ± 0.1	17.0 ± 0.3
[Hmim][PF_6_]	19.2 ± 0.5	28.9 ± 0.4	33.7 ± 0.2	17.8 ± 0.1
[Omim][PF_6_]	18.2 ± 0.2	30.7 ± 0.3	33.1 ± 0.3	18.0 ± 0.2
[Dmim][PF_6_]	17.9 ± 0.3	31.3 ± 0.2	32.5 ± 0.4	18.3 ± 0.1
[Emim][BF_4_]	20.5 ± 0.3	28.2 ± 0.5	34.9 ± 0.1	16.4 ± 0.4
[Bmim][BF_4_]	18.8 ± 0.1	30.1 ± 0.3	33.7 ± 0.2	17.4 ± 0.1
[Omim][BF_4_]	16.4 ± 0.1	31.2 ± 0.5	32.2 ± 0.3	20.2 ± 0.4
[OmPy][BF_4_]	15.6 ± 0.2	32.3 ± 0.3	32.0 ± 0.3	20.1 ± 0.4
[Bmim][Tf_2_N]	16.5 ± 0.3	33.0 ± 0.2	32.3 ± 0.2	18.2 ± 0.3
[Emim][TfO]	22.3 ± 0.6	28.2 ± 0.2	37.8 ± 0.1	11.7 ± 0.3
[Bmim][TfO]	24.9 ± 0.4	26.3 ± 0.3	39.3 ± 0.2	9.5 ± 0.1
[Bmim][CH_3_SO_3_]	25.3 ± 0.2	25.9 ± 0.4	40.3 ± 0.3	8.5 ± 0.2
[Omim][CH_3_SO_3_]	26.8 ± 0.4	26.0 ± 0.2	40.1 ± 0.2	7.1 ± 0.3
[Bmim][Cl]	25.3 ± 0.2	27.2 ± 0.3	39.0 ± 0.5	8.5 ± 0.2
[Emim][Cl]	25.7 ± 0.1	28.3 ± 0.3	38.3 ± 0.4	7.7 ± 0.5
[Omim][Cl]	26.3 ± 0.2	28.5 ± 0.4	38.2 ± 0.5	7.0 ± 0.3
[Bmim][NO_3_]	26.5 ± 0.3	28.3 ± 0.3	39.3 ± 0.1	5.9 ± 0.5
[Bmim][CH_3_COO]	26.3 ± 0.4	28.1 ± 0.3	39.1 ± 0.2	6.5 ± 0.1

Values given are the average of three replicates.
